# Fibrous Structures: An Overview of Their Responsiveness to External Stimuli towards Intended Application

**DOI:** 10.3390/polym16101345

**Published:** 2024-05-09

**Authors:** Mónica P. S. Ferreira, Afonso S. Gonçalves, Joana C. Antunes, João Bessa, Fernando Cunha, Raúl Fangueiro

**Affiliations:** 1 Fibrenamics-Institute for Innovation in Fiber-Based Materials and Composites, University of Minho, Campus de Azurém, 4800-058 Guimarães, Portugal; monicaferreira@fibrenamics.com (M.P.S.F.); afonsogoncalves@fibrenamics.com (A.S.G.); joaobessa@fibrenamics.com (J.B.); fernandocunha@fibrenamics.com (F.C.); rfangueiro@fibrenamics.com (R.F.); 2 Centre for Textile Science and Technology (2C2T), University of Minho, Campus de Azurém, 4800-058 Guimarães, Portugal

**Keywords:** responsive fibrous structure, weaving, knitting, electrospinning, stimuli-responsive polymer, temperature, light, pH

## Abstract

In recent decades, the interest in responsive fibrous structures has surged, propelling them into diverse applications: from wearable textiles that adapt to their surroundings, to filtration membranes dynamically altering selectivity, these structures showcase remarkable versatility. Various stimuli, including temperature, light, pH, electricity, and chemical compounds, can serve as triggers to unleash physical or chemical changes in response. Processing methodologies such as weaving or knitting using responsive yarns, electrospinning, as well as coating procedures, enable the integration of responsive materials into fibrous structures. They can respond to these stimuli, and comprise shape memory materials, temperature-responsive polymers, chromic materials, phase change materials, photothermal materials, among others. The resulting effects can manifest in a variety of ways, from pore adjustments and altered permeability to shape changing, color changing, and thermal regulation. This review aims to explore the realm of fibrous structures, delving into their responsiveness to external stimuli, with a focus on temperature, light, and pH.

## 1. Introduction

In the field of materials science and engineering, the quest for smart and responsive materials has spurred innovation across various domains. Within this paradigm, fibrous structures have emerged as versatile platforms, demonstrating remarkable adaptability to external stimuli. Fibrous structures like woven, nonwoven, knitted, and electrospun fibrous architectures have captured the attention of researchers, paving the way for a new era of responsive materials.

A fibrous structure refers to an assembly of fibrous elements, whether filaments or fibers, that together form a functional structure. These structures exhibit a thread-like or elongated form, spanning sizes from macroscopic to nanoscopic scales [1]. Relevant examples, considered in this review, include conventional textile-derived woven, knitted, and nonwoven fabrics, along with emergent fiber processing methodologies of the field, such as electrospun fibrous structures.

Woven fabrics are formed by interlacing or interweaving warp and weft yarns in directions perpendicular to each other, in a process known as weaving, the most popular technique of fabric formation [2]. Knitting, the second most widely used method of creating fabrics, generates knitted fabrics, which are made by forming loops in the width or length direction of the fabric [2]. Numerous variations in knitted fabrics exist, but they can be categorized into three fundamental groups of knitted structures [2]. All types of knitted fabrics stem from these foundational structures, which are: single-knit, rib, and interlock [2]. Woven and knit fabrics serve versatile functions across a wide range of industries, including fashion [3], biomedical [4], automotive [5], civil engineering [6,7], and filtration [8].

Unlike woven and knitted fabrics, nonwoven fabrics are crafted from fibers without limitations, created through the bonding or interlacing of fibers, or a combination of both [2]. This is achieved through mechanical, chemical, thermal, or solvent-induced methods, or a combination thereof [2]. Web-forming techniques, like drylaid system, wetlaid system and polymer-based system, and bonding techniques (thermal, chemical, and mechanical) are some of those methods [2]. Nonwovens serve diverse purposes, finding applications in durable or disposable products across various sectors such as personal care, health care, clothing, household items, automotive, construction, geotextiles, and filtration [2].

The properties of these fabrics can be influenced by: the type of fibers (cotton, polyester, etc.), the characteristics of the yarns (staple yarn, filament yarn, textured yarn, etc.), the fibrous architecture or structure (woven, knitted, etc.), and the application of chemical (such as water repellent finish, and flame retardant finish) and physical or mechanical (such as heat setting, shearing, and calendaring) finishing processes [2,9].

Electrospun structures, on the other hand, are composed of ultrathin fibers that are created through the electrospinning process. Electrospinning involves the application of a high voltage on a conductive polymeric solution of adequate viscosity [10]. The resulting electrospun structure is characterized by its nanometer to micrometer-scale fibers, exhibiting a high aspect ratio (ratio between length and diameter), high porosity, and small, but interconnected, pores [10,11,12]. The structure’s properties, such as porosity, surface morphology, and mechanical strength, can be tailored by adjusting the electrospinning parameters, including polymer concentration, solution flow rate, applied voltage, and spinning distance, among others [10,11,13]. Electrospun structures offer diverse applications, including filtration [14], thermal insulation [15], protective clothing [16], sensors [17], wound dressings [18] and tissue scaffolds [19].

Smart fibrous structures refer to structures created though the mentioned processes, either using smart fibers as raw materials, or by combining other smart materials with fibrous structures [20]. These structures possess functions of sensing, actuating, adapting, and communicating, allowing them to respond to environmental conditions or stimuli [21]. Examples include shape memory fibrous structures, which adjust their shape in response to stimuli, such as temperature, and color-changing fibrous structures that dynamically alter their color based on environmental changes. Thermoregulating fibrous structures offer functionalities such as thermal insulation and cooling, while waterproof and moisture-permeable structures provide breathable yet protective fabrics. Self-cleaning fibrous structures employ mechanism like rolling water droplets to maintain cleanliness. Additionally, electronic smart fibrous structures integrate technologies such as physiological sensors, enabling functionalities like safety monitoring [20].

This review seeks to investigate fibrous structures and their reactions to external stimuli, placing particular focus on temperature, light, and pH. The subsequent sections will discuss responsive fibrous structures, and responsive elements such as shape memory polymers, thermochromic materials, pH-responsive polymers, photothermal materials, and other responsive elements, elucidating their integration into fibrous structures to confer distinct responsiveness to each stimulus. The exploration will extend to the fabrication methods, exemplifying how these responsive elements can be incorporated into the fibrous structure. By examining the interplay between these stimuli and fibrous structures, this review aims to provide insights into the development of fibrous structures with tailored functionalities for applications ranging from self-regulating textiles that adapt to environmental conditions to membranes capable of adapting to surrounding conditions. Within the scope of this review, the goal is to synthesize insights from a wide range of literature sources, to provide a holistic understanding of the current state-of-the-art in smart fibrous structures. By elucidating the principles, applications, and integration methodologies of smart fibrous structures, this review seeks to fuel further research and innovation in this field.

## 2. Responsive Fibrous Structures

Responsive fibrous structures can sense and respond to a diverse array of external stimuli, including temperature [22], light [23], pH [24], humidity [25], electricity [26], magnetism [27], biological agents [28], chemical product [29] or pressure [29]. This adaptability is crucial as each stimulus provides a unique avenue for customizing structure properties and responses to specific needs. These structures find application across various domains such as wearable and smart textiles [24], water filtration [30], biomedicine [31], biomimetics [26], among others. In these applications, the fibrous structures exhibit versatile behaviors in response to environmental stimulus, showcasing functions like color changes [22], shape changes [32], self-cleaning processes [33], or perform other tailored responses.

As smart materials, responsive fibrous structures can be categorized into passive smart, active smart, or ultrasmart classifications based on their interactions with external stimuli [34]. Passive smart structures, such as those that are UV protective, antibacterial, or waterproof, sense the environment [34]. Active smart structures, like those with phase change materials and shape memory materials, sense and respond to environmental stimuli [34]. Ultrasmart structures go further, possessing cognitive and adaptative capacities, seen in applications like spacesuits and wearable computers [34].

The work by Šubrová et al. exemplifies a passive smart structure achieved through the treatment of cotton and polyester fabrics with formulations containing silver nitrate, zinc nitrate, and copper sulphate. This treatment effectively inhibits bacterial growth without actively responding to external stimuli [35]. On the other hand, Agra-Kooijman et al. demonstrated the effectiveness of an active smart structure by imparting temperature responsiveness to yarns and fabrics through the application of thermochromic paint. The treated samples exhibited a remarkable reversible thermochromic response, switching from red to blue as the temperature varied from 26 °C to 32 °C, and vice versa [22]. Wicaksono et al. presented an ultrasmart flexible textile platform for large-scale physiological sensing, capable of monitoring skin temperature, heart rate, and respiration. With stretchable knit electronics, it wirelessly tracks 30 skin temperature nodes, ensuring accuracy during various activities, showcasing its potential in wearable health monitoring [36].

### 2.1. Responsiveness to External Stimuli

Considerable research has been dedicated to creating fibrous structures with stimuli-responsive properties. These structures react to external triggers, that can be of physical, chemical, or biological nature [37]. Physical stimuli encompass factors such as temperature [38], light [23], pressure [29], magnetic [39] and electric [26] fields. Chemical stimuli involve responses to variations in pH [40] or exposure to specific solvents or substances [29], while biological stimuli include reactions to substances like glucose [41] or enzymes [42]. As a result, they undergo changes in properties such as swelling and porosity [43], color [38], and physical structure [44].

As it will be subsequently discussed, temperature, as a stimulus, enables dynamic changes in material properties and is the most well comprehended phenomenon [45]. On its turn, light is a noninvasive and precise external trigger [46], and pH is useful in applications where environmental pH variations can act as triggers. In this section, the focus will be on exploring the responsiveness of fibrous structures to temperature, light, and pH stimuli, and which elements can be integrated in those structures to make them responsive.

#### 2.1.1. Temperature

Of the numerous stimuli responses, the reaction to temperature stands out as the most extensively studied and well-understood phenomenon [45]. This is especially true for fibrous structures where temperature responsiveness plays a crucial role. For instance, in conventional textiles, while providing passive comfort, there is a lack of adaptability to changing environmental conditions and wearer activity levels [47]. Leveraging the extensive surface area of clothing textiles, they can be transformed into smart interfaces, offering benefits in managing temperature and moisture for the wearer [47], providing active thermoregulation [48]. Other example are filtration membranes, where temperature-sensitive materials can optimize the filtration efficiency by adjusting pore size in response to temperature changes [49]. Furthermore, in biomedical applications, fibrous structures designed to respond to temperature fluctuation can be used in drug delivery systems [50].

**Figure 1:**
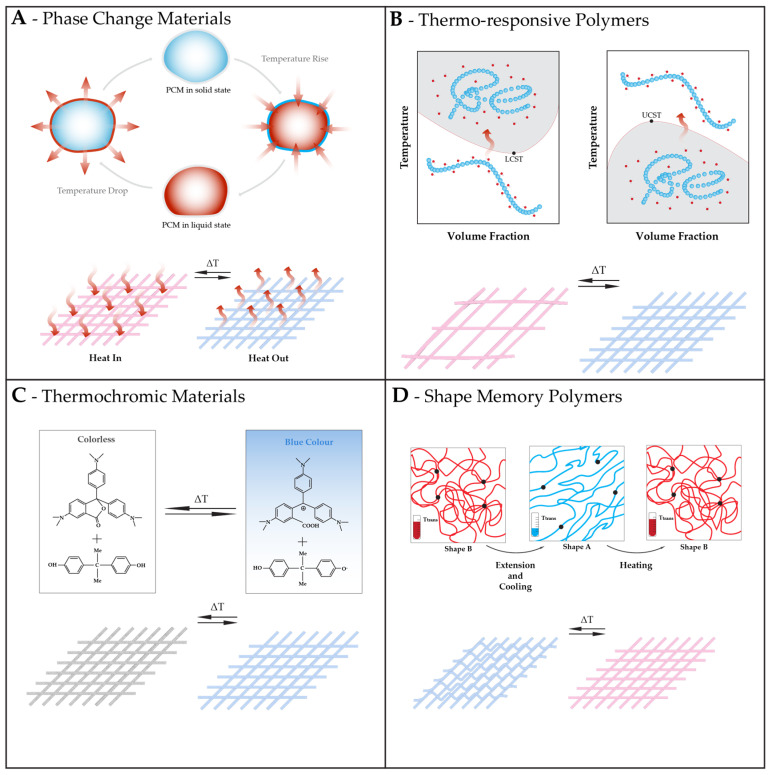
Examples of materials that can endow fibrous structures with responsiveness to temperature with their corresponding mechanism of action, and consequence in a responsive fibrous structure. (**A**) Phase change materials absorbing, storing, and releasing latent heat, with the same behavior occurring in a responsive fibrous structure. (**B**) Thermo-responsive polymers, with LCST and UCST behavior, transitioning between coil and globule states in solution with temperature variations, and consequent change in the porosity of a responsive fibrous structure. (**C**) Thermochromic materials (in the example, crystal violet lactone reacting with bisphenol A) changing color in response to temperature and giving the same ability to a structure containing them. (**D**) Shape memory polymers changing and recovering shape with temperature, with the consequent shape change in responsive fibrous structures.

This adaptability in fibrous structures can be achieved through the incorporation of materials that can sense temperature changes, materials capable of absorbing and/or releasing heat and materials that sense temperature changes and adjust fabric insulation by modifying breathability and moisture management [48]. Some examples of those materials include phase change materials (PCMs), shape memory materials (SMMs), temperature-responsive polymers, and thermochromic materials (TCMs), as seen in Figure 1.

##### Phase Change Materials

PCMs undergo a transition typically between solid and liquid states in response to temperature changes, allowing them to absorb, store, or release latent heat [51]. When the temperature surpasses the melting temperature (T_m_), the PCM absorbs heat, facilitating the melting process. Conversely, as the temperature decreases, the PCM undergoes crystallization at the crystallization temperature (T_c_), releasing the previously stored heat back into the surroundings [52]. This unique property makes them highly valuable for thermal energy management. Common examples of PCMs include linear chain hydrocarbons known as paraffin waxes (or *n*-alkanes), polyethylene glycol (PEG), fatty acids, and hydrated inorganic salts [48,51,53]. To address potential leakage challenges in their liquid state, PCMs are often encapsulated using microencapsulation, where they are enclosed in capsules to prevent direct contact with the environment [48,53]. PCMs are selected based on their specific phase change temperature, and they prove most effective when this temperature range aligns with the intended application. In textiles, for example, it is essential that their phase change temperature aligns with the human comfort range, typically between 15 and 35 °C [51]. PCMs that satisfy this criterion encompass paraffin waxes, predominantly consisting of straight-chain *n*-alkanes, and linear long-chain hydrocarbons like *n*-octadecane, *n*-hexadecane and *n*-eicosane [48,51]. PCMs play a crucial role in textiles [54], providing thermal regulation in clothing, as well as in building materials [55] and electronics [56] to control temperature.

An example of the successful incorporation of a PCM in a fibrous structure with potential application in wearable systems and protective fabrics is the work developed by Ma et al. The researchers used lauric acid, a 12-carbon chain fatty acid, in the creation of electrospun thermoregulated nanofibers. These nanofibers demonstrated phase change behavior, with a thermal storage capacity characterized by an enthalpy value of 103 J/g, equivalent to 72% of the pure lauric acid (69 J/g). To enhance the overall performance and functionality of the nanofibers, carbon nanotubes and zinc oxide (ZnO) particles were included in the fiber structure. This not only imparted UV resistance but also contributed to a high thermal conductivity (0.665 W/m.K). The integration of a polyvinyl alcohol (PVA) matrix ensured the nanofibers’ flexibility and robust tensile properties, adding to their versatility. Furthermore, a polydimethylsiloxane (PDMS) coating was applied, serving a dual purpose. It not only safeguarded the internal structure of the nanofibers but also conferred superior hydrophobicity and self-cleaning properties [57].

In another example, El Majd et al. investigated the use of a microencapsulated PCM (MPCM) in bio-based textiles for thermal insulation in building envelopes. The study focused on recycled cotton, hemp/linen/cotton, and Biofib Trio insulation materials modified with a commercial organic PCM. The MPCMs, with a melting temperature of 25 °C and latent heat of 195 J/g were applied using an acrylamide copolymer emulsion binder. The results indicated increased specific heat and thermal conductivity for the bio-based textiles, with recycled cotton showing the most cost-effective and environmentally friendly performance, with the specific heat capacity increased by 45% and 23% in the solid and liquid states, respectively. The study concludes that MPCMs enhanced thermal properties, which makes the material promising for energy-efficient building insulation [58].

##### Shape Memory Materials

Shape memory materials are a class of materials that can undergo a reversible transition between a temporary deformed state and a programmed shape when exposed to external stimuli [59]. Several materials exhibit the shape memory effect, most notably metal alloys, and polymers [59]. However, in recent years, attention has shifted away from metal alloys and towards shape memory polymers (SMPs). Compared to metallic shape-memory alloys, SMPs exhibit faster response times and greater deformability, low density, low cost, and ease in processing and in tailoring of properties [60,61].

In SMPs, unlike intrinsic properties, the shape-memory effect results from a combination of polymer morphology and specific processing, functioning as a polymer functionalization [62]. SMPs undergo a cycle of programming and recovery, initiated through conventional processes, like extrusion or injection molding [62]. This processing gives the polymer its permanent shape. Afterwards, the polymer is programmed by deforming and fixing it into a temporary shape [62]. Upon the application of an external stimulus, the polymer regains its initial permanent shape [62].

SMPs are elastic polymer networks containing molecular switches and netpoints. Netpoints, determining the polymer’s permanent shape, can be chemical (covalent bonds) or physical (intermolecular interactions) [62]. Physical cross-linking occurs in polymers with segregated domains, such as block copolymers [62]. The highest thermal transition temperature (T_perm_) domains act as netpoints (a hard segment), while the second highest thermal transition (T_trans_) domains serve as molecular switches (switching segment) [62]. When the working temperature exceeds T_trans_, the switching domains become flexible, resulting in elastic behavior above T_trans_ [62]. Deformation caused by external stress leads to the polymer snapping back to its initial shape when the stress is released [62]. To exhibit shape-memory functionality, the polymer must be temporarily fixed in a deformed state, achieved by reversible netpoints preventing recoiling [62]. Reversible netpoints can be physical (vitrification or crystallization) or covalent (functional groups attached to chain segments) [62]. Heat is commonly used to trigger the shape-memory effect, causing thermally induced cleavage of additional cross-links [62]. If based on physical interactions, a further distinction in T_trans_ can be made, which can be glass transition (T_g_) for amorphous polymers or melting temperature (T_m_) for semi-crystalline polymers [62,63]. Photo-reversible reactions of functional groups extend the shape memory technology to light stimuli [62]. SMPs can not only be activated by temperature [64] and light [65], but also by magnetism [66], electricity [63], moisture [67], pH value [68], and others.

Based on the reversibility of the shape memory effect, SMPs can be classified into two categories: one-way SMPs, that exhibit irreversible shape recovery, transitioning only from a temporary to a permanent shape; and two-way SMPs that allow reversible shape changes, enabling recovery of both the initial and temporary shapes with the introduction or interruption of the stimulus [69]. SMPs can be further categorized by the number of shapes involved in each memory cycle [70]. Dual SMPs involve one temporary shape transforming into a permanent shape [69]. Triple SMPs feature two temporary shapes in addition to the permanent one [69]. Multi-SMPs, capable of memorizing more than two temporary shapes, offer highly controllable recovery [69]. Among these, the most common SMPs is the dual SMPs [70]. SMPs have potential applications in smart textiles [64], biomedicine [71], self-healing composite systems [72], civil engineering [73], 3D printing [74] and other fields.

The most researched and attractive SMPs are polyurethanes (PU) [32,64,65,68,75]. Other SMPs commonly used include: poly(ε-caprolactone) (PCL) [71,76], which is a component of many PU materials; polyethylene terephthalate (PET) [26,76]; polylactic acid (PLA) [32] and co-polymers, such as poly(lactic-co-glycolic acid) (PLGA) [77]; polyethylene glycol (PEG) [78]; among others. In order to improve the recovery of the original shape, mechanical strength and reduced recovery time, for example, reinforcing fillers such as silica, carbon nanotubes, graphene oxides (GOs), and nanoclay, can be incorporated into SMPs [59]. Shape recovery (R_r_) and shape fixity rate (R_f_) describe the material’s ability to return to its original shape after undergoing deformation. R_f_ refers to a segment’s ability to correct or fix transient deformations during programming, while R_r_ denotes the material’s capability to recover to its original form afterward [59].

In Table 1 are some examples of shape memory polymers used in fibrous structures in recent years, along with their potential applications. The table highlights the variability in T_g_ and T_m_ among different polymers. For instance, PCUU has a T_g_ range of −13 °C to −18 °C and a Tm range of 48 °C–51 °C, whereas PLA exhibits a broader T_g_ range of 21 °C–60 °C and a higher T_m_ range of 142 °C–149 °C. The T_prog_ is influenced by factors such as T_g_ or T_m_, depending on the polymer’s amorphous or semi-crystalline nature, as well as the specific application.

An example of a fibrous structure with SMPs is the work from Choe et al., who developed a woven structure incorporating PLA and PU as SMP, complemented by silver nanowires (AgNWs) to confer asymmetric IR reflectivity and hydrophilicity. GO was also included for reinforcement. The fibers were created using wet spinning, which was followed by plain weaving and coating. The result was a textile with a hydrophobic bare polymer side and a hydrophilic AgNWs coated side, owing to the inherently hydrophobic nature of PLA and PU. The woven textile exhibited tunability in thermal insulation and asymmetric wettability through the deformation and recovery of its shape in response to stimuli. The degree of thermal insulation could be controlled, retaining 66% of the original value. Notably, the directional transportation of water droplets could be switched on/off based on the shape of the SMP textiles, which could be employed for sweat removal from the human skin [32].

##### Thermo-Responsive Polymers

To grasp the concept of thermo-responsive polymers, also known as temperature-responsive polymers, one must understand the notion of critical solution temperature. When an aqueous polymer solution is in a homogeneous state below a specific temperature and, above that threshold, separates into two distinct phases, rendering the polymer insoluble, it demonstrates a lower critical solution temperature (LCST) [83]. Conversely, if the reverse occurs—where a solution initially displaying two phases becomes a single phase upon heating, allowing the polymer to dissolve—the polymer exhibits an upper critical solution temperature (UCST) [83]. Notably, some polymers can showcase both phase transition behaviors [84]. These temperature-dependent changes are reversible and are associated with transitions between coil and globule states in solution [85], which are represented in Figure 1. The swelling behavior is attributed to the coil state with expanded hydrophilic chains, while the collapsed hydrophobic globule state indicates shrinkage [83,86]. This property makes temperature-responsive polymers valuable for applications like controlled drug release [87], food packaging [88], water filtration [30], and smart textiles [43].

The most well-known and researched thermo-responsive polymers are poly(N-isopropylacrylamide) (PNIPAM) [23,44,89,90] and its derivatives [91,92]. PNIPAM exhibits reversible phase transitions with an LCST at 32 °C, approximately, close to human physiological temperature [45]. Other widely used thermo-responsive polymers with LCST include: poly(N-vinylcaprolactam) PNVCL [93,94,95,96], poly(2-(dimethylamino)ethyl methacrylate) (PDMAEMA) [94], and poly(ethylene glycol) methyl ether methacrylate (PEGMA) [95].

Polymers with UCST in aqueous solution have received less attention in research compared to LCST polymers. Seuring and Agarwal [97] have reviewed UCST polymers and pointed out three main reasons for that: impractical observation conditions, such as extreme temperatures, high ionic strength, or low pH; sensitivity to electrolytes and concentration; and sensitivity to copolymer composition. Seuring and Agarwal [97] presented a detailed list of UCST polymers, some examples include: polyethylene oxide (PEO), poly(vinyl methyl ether) (PVME), polyvinyl alcohol (PVA), poly(acrylic acid) (PAAc) and poly(N-acryloyl glycinamide) (PNAGA).

In Table 2 are some examples of thermo-responsive polymers that have been used in fibrous structures, with their respective types (UCST or LCST) and applications. As it can be seen in the table, the different thermo-responsive polymers have LCST/UCST which, although different, are in very similar temperature ranges. The choice of polymer should consider the desired application. In addition, manipulation of the polymer, for example through co-polymerization with other polymers or monomers, can help adjust the temperature to the desired requirements for the desired applications.

Liang et al. utilized PNIPAM and PDMAPS to investigate textile yarns exhibiting opposite thermo-responsive wetting behaviors. By coating cotton yarns with polymer solutions through a dipping process, they created thermo-responsive yarns, suitable for weaving. The resulting smart yarns maintained qualities such as whiteness, biocompatibility, and washability. Woven textiles were then created, integrating both LCST and UCST yarns. The resultant hybrid textile demonstrated a dual functionality, providing a cooling effect (1.6 °C) and keep-warming effect (2.8 °C) in response to changes in the ambient temperature. This study introduced moisture/heat conditioning textiles with a broad temperature response range: LCST yarns from 25 °C to 47 °C, and UCST yarns from 22 °C to 54 °C. Furthermore, the yarns exhibited excellent wash resistance, exceeding 60 cycles [98].

Saadat et al. developed a two-step thermo-responsive nonwoven ultrafiltration membrane through the polymerization of lyotropic liquid crystals, employing the triblock copolymer PEO-PPO-PEO. This membrane exhibited distinct performance characteristics linked to the LCST of PEO-PPO-PEO and melting of PEO crystalline regions. At 35 °C, the LCST triggered changes in the membrane, leading to an increase in the molecular weight cutoff, transitioning from approximately 250 kDa (with a pore size of ~31 nm) at room temperature to 336 kDa (pore size ~37 nm). At 50 °C, additional modifications occurred, attributed to the melting of PEO crystalline regions. This brought about a further increase in the molecular weight cutoff to 570 kDa (pore size ~50 nm). These temperature-induced alterations contributed to increased membrane porosity, leading to improved hydration capacity, enhanced permeability, and improved cleaning efficiency, particularly in dealing with fouled membranes [49].

##### Thermochromic Materials

When subjected to temperature variations, thermochromic materials (TCMs) undergo a color change [106], which can be reversible [107] or irreversible [108]. This thermal phenomenon induces alterations in the material’s molecular structure, coinciding with shifts in its crystalline phase [106]. Based on the material properties and operating conditions, TCMs are generally classified into organic, inorganic, polymeric and hybrid [106]. Inorganic thermochromic materials exhibit thermochromic behavior from 70 °C to 500 °C [106], garnering interest in applications such as building construction. While stable above 200 °C, these materials may pose toxicity concerns and exhibit fixed transition temperatures [106]. Examples include Cr_2_O_3_-Al_2_O_3_ [109] and VO_2_ [110,111]. Organic thermochromic materials, like leuco dyes, undergo reversible color changes through structural alterations induced by heat, light, or pH changes [106]. Spiropyran leuco dyes, for instance, experience changes via UV-induced ring opening reactions [106]. Organic-inorganic hybrid thermochromic materials manifest color changes through structural phase transitions or intermolecular interactions [106]. Polymeric thermochromic materials encompass various types, including those with inherent thermochromism, those embedding thermochromic pigments, those with thermo-responsive additives, and those containing thermo-responsive dyes [106]. Examples range from liquid crystalline polymers and conjugated polymers to polymers with microencapsulated leuco dye systems [106].

In inorganic thermochromic compounds, reversible color changes often stem from temperature-induced alterations in crystal symmetry during reversible phase transitions [106]. This transformation is linked to changes in ligand geometry or coordination number, impacting the crystal field energy within the visible electromagnetic radiation range [106]. On the other hand, organic and polymeric thermochromic materials undergo color changes due to variations in molecular structure, affecting the energy levels of the highest occupied molecular orbital (HOMO) and lowest unoccupied molecular orbital (LUMO) [106]. Additionally, the interconversion of stereoisomeric forms in organic molecules contributes to thermochromism [106].

Several thermochromic materials face challenges such as high cost, operational temperature limitations, durability, or manufacturing complexities, rendering them impractical [112]. Leuco dye-based materials, being cost-effective and easy to produce, are a preferred choice for various commercial applications, including the manufacturing of thermochromic inks [112,113].

TCMs have great potential applications in aerospace [110], inks [114,115], anticounterfeiting [114,115], textiles [116], sensors [109], smart windows [117], and pavements [118].

Recognizing the limited color-change range inherent in common thermochromic materials, Zhou et al. sought to address this constraint by expanding the color-change range of organic thermochromic materials for everyday textiles. They developed a novel mixed-colorant thermochromic core material, incorporating crystal violet lactone (CVL) as the color former, bisphenol A (BPA) as the developer, solvent-based dyes as extended dyes, and dodecyl dedecanoate as the solvent. The researchers synthesized mixed-colorant thermochromic microcapsules (MCTMs) through in situ polymerization, incorporating formaldehyde-melamine resin as the shell material. Various analyses revealed that MCTMs exhibited high thermal stability, with an encapsulation rate of 87%. The MCTMs were utilized in printing paste for cotton fabrics, demonstrating reversible color changes across a spectrum of tones within the ambient temperature range of 20–35 °C. The MCTMs, integrated into printed fabrics, exhibited high reliability, durability, and vibrant colors, showcasing their potential for applications in thermochromic intelligent textiles [119].

Sahebkar et al. pointed out that incorporating encapsulated leuco dyes with printing pastes, leads to dilution of the color and shortened longevity. To address this, the researchers turned to electrospinning as a method for creating thermochromic fibers, enabling the integration of the dye into the fabric. Their approach involved spinning single strands of poly(methyl methacrylate) (PMMA), a commercial black thermochromic dye, and a solvent (either N-dimethylformamide (DMF) or chloroform). The resulting fibers exhibited a distinct color transition from grey to white, presenting a color change around 30 °C. The study delved into the impact of using different solvents (DMF and chloroform) on the fabrication of thermochromic fibers and found that chloroform yielded larger diameter fibers with clearer color contrast and more pronounced phase change properties. This study successfully demonstrated an inexpensive and versatile technique to fabricate thermochromic fibers [112].

#### 2.1.2. Light

Light emerges as a cost-effective, safe, and readily available energy source, setting it apart from alternative stimuli such as temperature, pH, and enzymes [46]. A noteworthy advantage of light lies in its noncontact nature when instigating responsive behaviors [46]. The precision in controlling photo-responsive structures is achieved with high spatial and temporal resolution by manipulating parameters like wavelength, intensity, exposure area, and irradiation time of incident light [46]. Various light wavelengths, including ultraviolet (UV), visible, and near-infrared (NIR), are commonly harnessed as energy sources [46].

**Figure 2:**
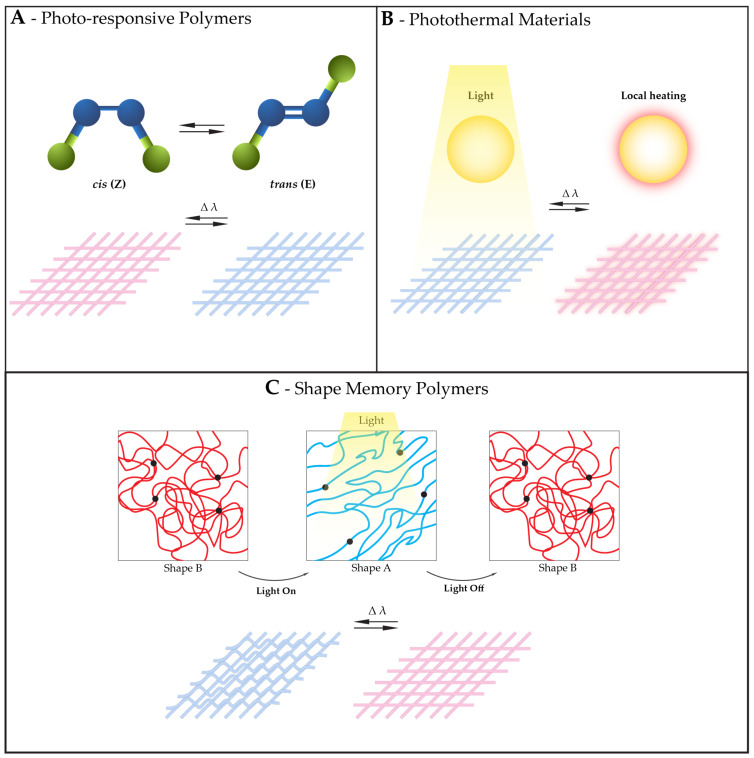
Examples of materials that can endow fibrous structures with responsiveness to light with their corresponding mechanism of action, and consequence in a responsive fibrous structure. (**A**) Photo-responsive polymers. In the example, a molecule suffers reversible isomerization when exposed to a specific light, which results in a change in color in the responsive fibrous structure. (**B**) Photothermal materials converting light into heat, increasing the temperature in a fibrous structure exposed to light. (**C**) Shape memory polymers changing and recovering shape when exposed to light, with the consequent shape change in responsive fibrous structures.

Photo-responsiveness can impart benefits to a fibrous structure such as the ability to change the porosity of the structure in the presence of light, which can be useful, for instance, to enhance the cleaning efficiency of membranes [120], prolonging their life cycle. Other examples of benefits include color changes for aesthetic or functional purposes [121] or light-triggered drug delivery [122]. To give fibrous structures the capacity of responding to light, photo-responsive polymers, photothermal materials or SMPs can be integrated in the structures, as represented in Figure 2.

##### Photo-Responsive Polymers

Photo-responsive polymers, also known as light-responsive polymers, can undergo physical or chemical changes in response to a light stimulus [83]. The induced molecular changes can manifest macroscopically in material properties, such as shape, wettability, solubility, optical characteristics, conductivity, and adhesion [123]. The efficacy and potential applications of photo-responsive polymers depend on three key parameters: the magnitude of property change post-light triggering, the speed of the change, and the reversibility of the process [123]. Reversible reactions may involve processes like isomerization or conformational changes, while irreversible reactions could include cross-linking or degradation. To create a photo-responsive polymer, a photo-responsive functional group or chromophore must be incorporated into the polymer chain [123]. The selection of the specific photo-responsive moiety is crucial, as it determines the nature of the photochemical reaction upon light irradiation [124]. For instance, moieties like *o*-nitrobenzyl esters may undergo an irreversible transformation, while others, such as azobenzenes, can react reversibly [124]. Careful consideration of both the nature of the photochemical reaction and the positioning of the photo-responsive moiety allows for the tailoring of polymers with specific and desired light-responsive properties for diverse applications [124].

Photo-responsive polymers can be broadly categorized into photochemical or photothermal polymers [83]. The photochemical effect involves light-induced moieties’ reactions, such as isomerization and dimerization [83]. Azobenzenes, especially, undergo trans-cis isomerization under UV light and cis-trans isomerization under visible light [83]. Photochromic polymers, which undergo changes in color upon exposure to light, are included in the photochemical category. Photothermal polymers can convert light into heat [83]. Examples include polydopamine, polypyrrole, and polyaniline [83].

Chen et al. adopted a bioinspired approach, utilizing catechol-functionalized azobenzene-containing copolymers, specifically poly(DMA-co-FAzo-co-FMA), inspired by mussel foot proteins. Synthesized through solution radical polymerization, these copolymers were applied via dip-coating to modify cotton fabrics and melamine sponges. The resulting smart surfaces exhibit photo-switchable wettability, displaying rapid reversible transitions between high hydrophobicity and superhydrophilicity under alternating UV and visible light. This behavior is attributed to the photo-triggered *cis*-*trans* isomerization of the azobenzene groups in the azo-copolymers. This bioinspired strategy opens avenues for large-scale production of superwetting surfaces, holding potential applications in microfluidic devices, biomedicine, and sensors [125].

Miao et al. focused on creating a self-cleaning photothermal superamphiphobic fabric. This involved the self-polymerization of dopamine (DA) into polydopamine (PDA), on a cotton fabric surface, serving as a secondary reaction platform. In situ growth of silicon dioxide (SiO_2_) nanoparticles on the PDA@fabric surface created micro–nano rough structures. The SiCO_2_ surface was further treated with a fluorosilane to reduce the fabric’s surface energy, resulting in superamphiphobic properties, characterized by water and oil contact angles exceeding 150°. The resultant fabric demonstrated resistance to various pollutants, chemical solutions, UV radiation, high temperatures, and cyclic washing. Additionally, under simulated sunlight, the fabric’s surface temperature can rise from 20 °C to 37 °C, showcasing its photothermal capabilities. This multifunctional cotton fabric demonstrates potential applications in self-cleaning and photothermal conversion [126].

##### Photothermal Materials

Carbon nanomaterials like carbon nanotubes (CNTs), graphene, and graphene oxide (GO) are commonly used for preparing photothermal materials due to their high absorption coefficients across the UV-visible to NIR regions [127]. Challenges in their applications, such as poor water dispersibility and aggregation tendency, can be overcome by surface modification with polymers [127]. PEG is a widely used polymer for this purpose, enhancing dispersibility and stability [127,128].

Metal and metal compounds, such as Au nanomaterials, demonstrate the capability of generating heat under light irradiation due to localized surface plasmon resonance (LSPR) [127]. LSPR is a phenomenon related to the interaction of light with metal nanoparticles (NPs) [129]. When metallic NPs, particularly noble metals, such as Au and Ag [130], are exposed to light, the electrons on the metal surface can collectively oscillate in response to the incident electromagnetic field [129]. When the frequency of the incident light is close to the natural frequency of the nanoparticles, surface plasmon oscillations occurs [131]. The surface plasmon frequency is highly sensitive to the size, shape, and composition of the nanoparticles, as well as the surrounding medium [129,130]. The resonance leads to enhanced light absorption and scattering by the nanoparticles. This phenomenon improves the photothermal conversion capability of the nanoparticles, making them more effective in converting light into heat [132].

Challenges like long-term toxicity and aggregation in aqueous media can be addressed by modifying these nanomaterials with polymers like PEG and polyelectrolytes, improving biocompatibility and suppressing cytotoxicity [127].

Ramesh et al. developed the first composite nonwoven fiber mats (NWF) with IR light-controlled permeability, utilizing PNIPAM, as a thermo-responsive polymer, and graphene oxide nanoparticles (GONPs), as photothermal materials. When exposed to IR light, the GONPs convert it into localized heat, causing the contraction of PNIPAM-based microgels embedded in the pore space of the NWFs. This reversible contraction, occurring above the LCST of PNIPAM, enables control over the membrane’s porosity, and, consequently, its permeability. The study identified key design parameters influencing permeability control, specifically the monomer composition of the microgels and the GONP-to-microgel ratio. In contrast, control NWFs coated with GONP-free microgels only exhibited thermal responsiveness, while native NWFs displayed no smart-gating behavior. This approach provides a versatile and responsive means of regulating membrane permeability with potential for processing temperature-sensitive bioactive ingredients or remote-controlled bioreactors [23].

Zhu et al. focused on improving the comfort of cotton fabrics by utilizing the LSPR effect through densely packed hybrid nanogels containing Au NPs. These nanogels, synthesized though emulsion polymerization, exhibited a linear shrinkage behavior upon temperature increase due to the thermo-responsive components. The addition of Au NPS enhanced their light-responsive and photothermal conversions capabilities. When cross-linked onto cotton fabrics and exposed to visible light irradiation, the hybrid nanogels exhibited a significant increase in moisture permeability. This enhancement is attributed to the LSPR effect inducing efficient photothermal conversion and substantial shrinkage of the hybrid nanogels. Additionally, the cotton fabrics with hybrid nanogels, due to the presence of Au NPS, efficiently shield incident light, demonstrating good heat insulation. The dual responsiveness to light and temperature makes these fabrics promising candidates for applications in smart textiles, offering improved moisture permeability and thermal insulation in various scenarios, such as outdoor activities and daily wear [132].

#### 2.1.3. pH

**Figure 3:**
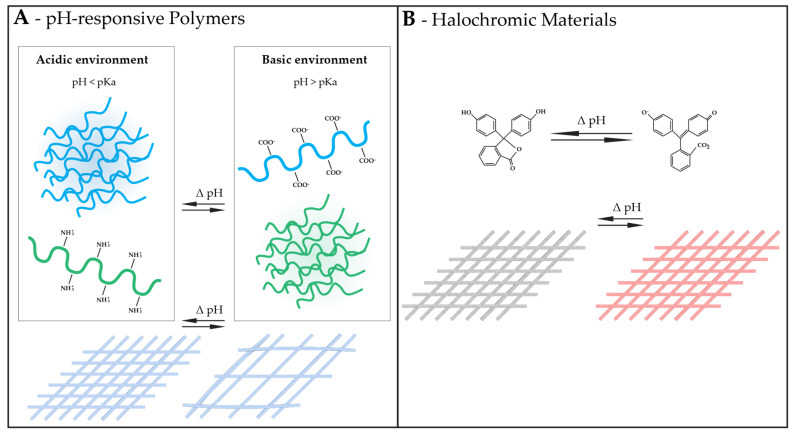
Examples of materials that can endow fibrous structures with responsiveness to pH with their corresponding mechanism of action, and consequence in a responsive fibrous structure. (**A**) pH-responsive polymers changing hydrophilicity with pH, leading to a porosity change in a fibrous structure. (**B**) Halochromic materials. In the example, a pH variation results in a ring opening of a phenolphthalein halochromic dye, resulting in a color change in a fibrous structure.

The significance of pH responsiveness in fibrous structures lies in its capacity to impart dynamic functionalities. Fabrics adjusting properties like color [133], wettability, or porosity [47] based on surrounding pH improve user experience. From health care applications like wound care and drug delivery to addressing environmental concerns through efficient oil–water separation and responsive filtration, the relevance of pH responsiveness extends to optimizing performance and functionality in various contexts [40,134,135,136]. To impart fibrous structures with pH responsiveness, pH-responsive polymers or halochromic materials, for example, can be employed (Figure 3).

##### pH-Responsive Polymers

pH-responsive or pH-sensitive polymers are a type of stimuli-responsive polymers that undergo physical and chemical changes based on the pH of their environment [83,137]. These polymers contain weak acidic or basic groups in their structure, allowing them to accept or release protons depending on the pH [83,137]. The resultant pH-dependent transitions offer a means of precisely adjusting hydrophilicity, leading to various effects like precipitation/solubilization, swelling/deswelling of hydrogels, and changes in the hydrophobic/hydrophilic characteristics of surfaces and particles [137]. Within this classification, pH-responsive polymers can be broadly categorized into two types. Firstly, there are pH-responsive acidic polymers that incorporate weak acidic groups, such as carboxylic, sulfonic acid, phosphonic acid and boronic acid groups [137]. Polyacrylic acid (PAA) [40] and poly(2-acrylamido-2-methylpropanesulfonicacid) (PAMPS) [138] are examples of pH-responsive polymers containing weak acidic groups, more specifically carboxylic and sulfonic acid groups, respectively. Secondly, pH-responsive basic polymers are characterized by the presence of weak polybases with amine groups in their side chains, or polymers containing tertiary amine, pyrrolidine, morpholino, or pyridine groups, among others [137]. Poly(2-dimethylaminoethyl methacrylate) (PDMA) [139] and poly(N-ethylpyrrolidine methacrylate) (PEPyM) [140] are illustrative instances of pH-responsive polymers that incorporate basic group, with PDMA featuring a tertiary amine structure and PEPyM incorporating pyrrolidine groups [137]. Natural polymers, including dextran, hyaluronic acid, alginic acid, chitosan, and gelatine, also find practical application in pH-responsive systems [137]. Additionally, certain polymers exhibit responsiveness to multiple factors, or they may be designed as copolymers combining different monomers to display diverse stimuli-responsive behaviors, such as simultaneous sensitivity to both temperature and pH conditions [137].

pH-responsive polymers find applications mainly in drug delivery systems [141], smart membranes [142], biomedical [143] and environmental [144] applications.

Table 3 shows some examples of pH-responsive polymers and co-polymers that have been used in fibrous structures, with the group that gives them their responsiveness, and applications.

Liao et al. developed a pH-responsive nanofibrous mat, using electrospinning, containing P(DEAEMA-co-MMA-co-ABP) for drug release. In acidic conditions, the copolymer swelled, the nanofibers absorbed water and became hydrophilic, releasing encapsulated substances. In alkaline conditions, the copolymer deswelled, becoming hydrophilic, which caused the fibers to contract and retain the encapsulated substances. In practical terms, when loaded with amoxicillin, the nanofibers showed a significant release of the drug (around 40% at pH 5.8 and 35% at pH 6.4), in contrast to a reduced release of the drug at pH 7.4 and pH 8.5 (12% and 8%, respectively) after 84 h [31].

As previously mentioned, certain polymers or copolymers are designed to exhibit responsiveness to multiple factors, one example is P(NIPAM-co-MAM), responsive to both temperature and pH. Genç and Aksoy applied this copolymer to a cotton weaved fabric using a double-bath impregnation method. The resulting fabric demonstrated reversible thermo-responsive wetting properties, transitioning from hydrophilic to hydrophobic states based on temperature changes. The fabric also exhibited pH-responsive water absorption and could modulate water vapor permeability by adjusting pore size and hydrophilic character with temperature variations. The pH sensitivity of the copolymer arose from the presence of basic amino groups in the chemical structure of the MAM monomer. The fabric, when subjected to different pH conditions, showed a pH-sensitive uptake property. Specifically, water uptake values decreased at basic pH, which exceeded the pK_b_ value of the MAM monomer. This decrease was attributed to the diminishing hydrophilicity of the copolymer molecules at higher pH levels compared to acidic and neutral pH values [47].

##### Halochromic Materials

Halochromic dyes or materials are a class of substances characterized by their ability to manifest a reversible change in color in response to changes in pH variations [146]. This phenomenon is tied to the protonation or deprotonation of the compounds, prompting consequential structural changes. These modifications, in turn, lead to alterations in the absorption or reflection properties of the compounds, thereby causing reversible changes in the perceived color [147].

Halochromic dyes can be broadly categorized into various classes. The primary categories of commercially significant pH-sensitive dyes include phthalides, triarylmethanes, and fluoranes [148]. Methyl yellow, bromothymol blue [149], methyl orange [150], phenol red [151] and bromocresol purple [152] are some examples of halochromic synthetic dyes.

Synthetic dyes, like the crystal violet triarylmethane dye, can pose significant risks to the environment and human health [153,154]. As environmental safety awareness grows, there is a heightened focus on researching natural colorants that are biodegradable. One notable example is anthocyanins, which are extracted from red cabbage [155]. In a study by Devarayan and Kim, electrospun cellulose nanofibers were functionalized with this pigment to create an eco-friendly and reversible pH sensor. The sensor could detect pH values from 1 to 14, displaying a distinct color for each pH. The research highlights potential applications in health monitoring [155].

Halochromic materials are also useful in wound management. Wounds undergo pH changes during different stages of the healing process. For example, infections can elevate the pH, while healing is associated with a decrease in pH [156]. Halochromic materials allow for visualizing these pH changes, providing a simple noninvasive method to monitor the wound status or detect an infection [157].

Atav et al. applied bromcresol purple to a cotton knitted fabric using conventional exhaust and padding methods, aiming to optimize the dyeing process. The fabrics exhibited significant color-changing ability in response to pH variations with both acid-base solutions and vapours. In general, the fabric is yellow in neutral state and its color turns into orange in acidic and into green-dark blue in basic conditions. Although both exhaust and padding methods were deemed suitable for halochromic dyeing, the study highlights the need for further research to improve fastness values. The application of bromcresol purple for creating small sensors, such as garment tags or wristbands, is suggested, emphasizing functionality in detecting acid/alkali vapor dangers [148]. Another example of a pH sensor is that developed by Leite et al. The researchers devised a pH sensor designed to function as a patch strategically positioned within chemical protective clothing. The sensor incorporates pH indicators such as bromothymol blue, methyl red, methyl red sodium, methyl orange and bromocresol purple. Theses sensors demonstrated visible responses upon exposure to acidic and alkaline substances, whether in liquid or gaseous form [158].

**Figure 4:**
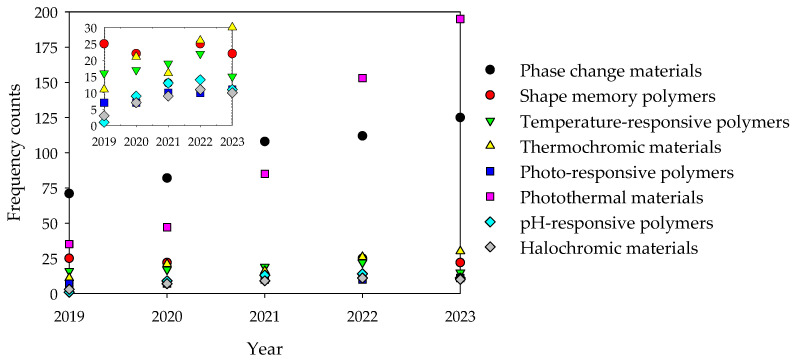
Approximate frequency counts, within published literature of the last five years (2019–2023) of the usage, in fibrous structures, of different responsive materials, namely: phase change materials, shape memory polymers, temperature-responsive polymers, thermochromic materials, photo-responsive polymers, photothermal materials, pH-responsive polymers and halochromic materials (database: Scopus).

Figure 4 depicts the recent trends in the utilization in fibrous structures of the different responsive materials addressed in the review. The data are derived from an analysis of approximate frequency counts within the published literature spanning the last five years (2018–2023), sourced from the Scopus database. Initially, PCMs held the highest usage rates. However, their dominance was surpassed by photothermal materials in 2022 and 2023, which had previously ranked as the second most utilized. Over the past few years, there has been a notable surge in the utilization of these materials, with photothermal materials experiencing particularly pronounced growth. Conversely, the least utilized materials include pH-responsive polymers, halochromic materials and photo-responsive polymers. Despite overall growth in utilization across all materials, exceptions include SMPs and thermochromic materials, both experiencing a decline in usage in 2021.

## 3. Production of Responsive Fibrous Structures

A variety of techniques are employed to create responsive fibrous structures, involving the incorporation of responsive elements during, or after, the manufacturing process of the structure. Each method offers unique advantages, with the incorporation of the responsive elements at the level of the fiber, yarn, or structure.

At the fiber level, methods like electrospinning, wet-spinning, and melt-spinning come into play. In electrospinning, responsive polymers or nanoparticles can be incorporated into the spinning solution, yielding fibers inherently responsive to external stimuli. For instance, Williams et al. [38] blended thermochromic liquid crystals and polycaprolactone or polystyrene in chloroform, creating thermochromic electrospun nonwoven mats. Similarly, Ma and colleagues [57] produced phase change nanofibers through electrospinning, incorporating lauric acid (LA) as PCM, PVA as a supporting material, sodium dodecyl sulphate (SDS) as an emulsifier, and ZnO@MWCNT for thermal conductivity. Electrospinning solutions containing chitosan and pectin were also explored for pH-responsive nanofibrous films by Celebioglu et al. [159]. Melt-spinning involves extruding molten polymer to form fibers. Fibers with responsive properties can be obtained by incorporating polymers or additives into the melt. He et al. [160] developed thermo-responsive woven structures using fibers containing microencapsulated PCMs, which were incorporated during wet spinning. In another example, Gupta et al. [161] produced a SMP filament via melt spinning, using PHA, MDI (4,4′-diphenylmethane diisocyanate) and BDO (1,4-butanediol). This filament was combined with polyester (PES) yarns to produce knit fabrics, which were subsequently submitted to shape memory programming, by heating, deforming, cooling, and reheating. Melt spinning was also the technique used by Khadse and colleagues [162], but, in this case, the researchers did not incorporate responsive or shape memory polymers. Instead, they produced bicomponent fibers using resins with different coefficients of thermal expansion, Hytrel and Crastin, which were then used to produce self-crimping thermo-responsive nonwoven battings. On the other hand, wet spinning involves the extrusion of a polymer solution into a coagulation bath, with the polymer solidifying into fibers. Like melt spinning, the incorporation of responsive polymers or additives can endow the fibers with responsiveness. Choe and team [32] applied this technique to make fibers using PLA, PU, GO and DMF as solvent. The PLA/PU/GO fibers were later weaved into a SMP textile, which was programmed to obtain the shape memory behavior.

At the level of the fiber, yarn and fabric, techniques like impregnation and coating can be employed. Dip-coating is a method where the substrate is immersed in a solution that can contain responsive materials, facilitating their absorption or adherence to the substrate. Agra-Kooijman et al. coated 100% PES yarns with SFXC and Hallcrest, which are liquid crystal inks, by looping the yarns through pulleys and ink barrels, after which they weaved and knitted the yarns, resulting in thermochromic fabrics [22]. Liang and team [98] prepared LCST polymer coating solutions by mixing NIPAM, ethylene glycol dimethacrylate (EGDMA), and initiator (2,2-diethoxyacetophenone (DEAP)) in ethanol; and UCST polymer coating solutions by mixing N,N-dimethyl (methacryloylethyl) ammonium propane sulfonate (DMAPS), EGDMA and 2-hydroxy-4-(2-hydroxyethoxy)-2-methylpropiophenone (Irgacure D-2959)) in water/trifluoroethanol. These coating solutions were used for dipping cotton yarns, resulting in LCST and UCST yarns, which were later used for weaving, culminating in textiles with yarns with opposing thermo-responsive behavior. Similarly, spray coating can be used to evenly distribute a responsive agent-containing spray over a substrate, ensuring uniform coverage. Lin and team [105] used this technique to coat one side of a knitted cotton fabric with a solution containing a thermo-responsive polymer (SBMA), N,N’-methylenebis(acrylamide) (MBA) and lithium phenyl(2,4,6-trimethylbenzoyl)phosphinate (TPO-Li). The researchers subsequently covered the coated fabric with a tape mask with hole patterns, followed by UV cross-linking, resulting in a fabric with channels that can switch mode depending on the ambient temperature. Impregnation can also be used to saturate or permeate a material with responsive agents, offering a convenient means to introduce responsiveness without complex processing steps. A noteworthy example of the application of this technique is a work developed by Genç et al. [47], who created a fabric with dual responsiveness, to pH and temperature. The authors synthesized P(NIPAM-co-MAM) through free radical addition polymerization and applied this copolymer to cotton fabrics using a double-bath impregnation. The first bath contained the BTCA (1,2,3,4-butanetetracarboxylic acid) cross-linker and the SHP (sodium hypophosphite) catalyst, and the second bath contained the aqueous polymer solution. In another study, from the same authors [43], PHEVAH was obtained through free radical polymerization, and the same double bath impregnation method was used to apply the polymer solution to a cotton fabric.

Alongside the techniques outlined above, there are other methods used to make responsive structures. Saadat et al. [49], for instance, prepared nonwoven thermo-responsive membranes by casting lyotropic liquid crystals (LLC) on nonwoven polyester sheets. The LLC were prepared with PEO-PPO-PEO, ammonium persulfate (APS), n-butyl acrylate (nBA) and EGDMA. In another example, Iqbal and colleagues [163] took advantage of the natural warmth and thermal insulation of wool fibers. Their work focused on descaling raw wool fibers using nanoparticles of calcium carbonate, sodium hypochlorite, and hydrochloric acid, and production of different knit structures using double-plied ring-spun dyed wool yarns with shape memory effect. Yang and associates [101] prepared a thermo-responsive cotton fabric by horseradish peroxidase (HRP)/acetylacetone (ACAC)/hydrogen peroxide (H_2_O_2_)-initiated ‘‘graft from’’ polymerization on the fiber surface, with MEO_2_MA and OEGMA_500_ as thermo-responsive monomers. Qi et al. [99] used the same approach to develop thermo-responsive textiles for oil–water separation, but PDMAPS was used as thermo-responsive polymer instead.

As mentioned above, multiple techniques have been applied in the construction of responsive fibrous structures. Many of them are based on the integration of responsive elements, like PCMs, SMPs and responsive polymers [30,32,49,105,160], while few make use of the properties of materials to endow the structures with the capacity to respond to stimuli [162,163]. Nevertheless, it is evident that the interest in SMPs and responsive polymers is increasing, while PCMs and chromic materials already appear to be more established. In essence, the methods employed in the production of responsive fibrous structures are bespoke to the intended applications and the properties and materials required. 

Table 4 summarizes recent examples of responsive fibrous structures from the literature, along with the methodology of fabrication and potential application.

## 4. Conclusions

The interest in responsive fibrous structures, as smart materials, has been gaining popularity in recent decades. Fibrous structures, such as woven, nonwoven, knitted, and electrospun structures, when endowed with responsivity, find enhanced value in applications across diverse industries, including fashion, biomedical, automotive, civil engineering, and filtration technologies.

Responsive fibrous structures are capable of sensing and adapting to external stimuli. These stimuli span a spectrum encompassing temperature, light, pH, humidity, electricity, magnetism, biological agents, chemical products, and pressure. While temperature responsiveness, facilitated by materials such as PCMs, and thermochromic materials, is a well-explored path in fibrous structures, other stimuli like light and pH present promising opportunities for further innovation. As temperature-responsive elements, PCMs and thermochromic materials have already made significant strides in industrial applications. However, this review has illustrated the vast potential of SMPs and thermo-responsive polymers for integration into fibrous structures. While SMPs offer a significant promise, for applications in smart textiles, for example, their utilization is somewhat constrained by the requirement for relatively high temperatures to trigger the shape memory effect.

Despite the progress made so far in responsive fibrous structures, the processes and materials still need further development. In the quest to develop the area, it becomes imperative to enhance the adaptability of these processes and materials for industrial settings. The transition to industrialization is crucial, requiring efforts to ensure scalability and optimize efficiency for large-scale production. While electrospinning finds extensive use in research, it is not yet fully industrialized. Conversely, techniques like weaving, knitting, impregnation, and coating are already entrenched in industry, with their lack of industrialization for these purposes attributed to different factors. In the case of thermo-responsive polymers, for example, their utilization necessitates a synthesis phase typically involving polymerization. Since these polymers are not readily available for such applications, developing synthesis strategies becomes imperative to streamline industrial production, particularly for application like coating techniques. In the future, it may also be of interest to improve the multifunctionality of responsive fibrous structures. While ongoing investigation delve into materials responding to multiple stimuli, this aspect remains relatively unexplored in the context of fibrous structures. Furthermore, in emerging advances, emphasis should extend beyond the sole consideration of the functionality of responsive fibrous structures. It is crucial to broaden the scope and concurrently prioritize the sustainability of both materials and processes integral to their development.

## Figures and Tables

**Table 1:** Shape memory polymers in fibrous structures: recent examples from literature (2021–2024) with their applications, glass transition temperature (T_g_, °C), melting temperature (T_m_, °C) programming temperature (T_prog_, °C), shape-recovery (R_r_, %) and shape-fixity ratio (R_f_, %).

Polymer	T_g_ (°C)	T_m_ (°C)	T_prog_ (°C)	R_r_ (%)	R_f_ (%)	Applications	Ref.
PCUU	−13–18	48–51	50	99	96	Tissue engineering	[79]
PLA	21–60	142–149	40–60	61–100	74–100	Smart textiles	[80]
PLA/PU	55	-	80	-	-	Smart textiles	[32]
PU	45	-	80	91–98	76–80	Smart textiles	[64]
	45	149	>45	99	-	Intelligent structures	[81]
	65	-	70	50–99	93–99	Smart textiles	[82]

PCUU: poly(carbonate-urea-urethane).

**Table 2:** Thermo-responsive polymers/co-polymers in fibrous structures: recent examples from the literature (2021–2024) with their types, transition temperature (T_trans_, °C) and potential applications.

Polymer	Type	T_trans_ (°C)	Applications	Ref.
PDMAPS	UCST	33	Smart textiles	[98]
		28	Smart oil–water separation fabric	[99]
PEO–PPO–PEO	LCST	35	Ultrafiltration membrane	[49]
		29–98	Forward osmosis membrane	[100]
PHEVAH	LCST	34	Smart textiles	[43]
P(MEO_2_MA-co-OEGMA_500_)	LCST	29	Smart textiles	[101]
PNIPAM	LCST	39 (VPTT)	NWFs for processing temperature-sensitive bioactive ingredients or for use in remote-controlled bioreactors	[23]
		32	Thermo-responsive membrane	[30]
		32	Smart textiles	[98]
		31–32	Smart textiles	[102]
		31	Smart textiles	[103]
P(NIPAM-co-AAm)	LCST	33–37	Smart membrane for water treatment	[104]
P(NIPAM-co-MAM)	LCST	33–41	Smart textiles	[47]
SBMA	UCST	26–27	Smart textiles	[105]

PDMAPS: poly[N,N-dimethyl (methacryloyl ethyl) ammonium propane sulfonate]; PEO–PPO–PEO: poly[(ethylene oxide)-block-(propylene oxide)-block-(ethylene oxide)]; PHEVAH: poly(2-hydroxyethyl-6-(vinyl amino)hexanoate); P(MEO_2_MA-co-OEGMA_500_): diethylene glycol monomethyl ether methacrylate-co-poly(ethylene glycol) methyl ether methacrylate; P(NIPAM-co-AAm): poly(N-isopropylacrylamide-co-acrylamide); NWFs: nonwoven fiber mats; P(NIPAM-co-MAM): poly(N-isopropylacrylamide-co-methacrylamide); SBMA: [2-(methacryloyloxy)ethyl]dimethyl-(3-sulfopropyl); VPTT: volume phase transition temperature.

**Table 3:** pH-responsive polymers/co-polymers in fibrous structures: recent examples from literature (2021–2024) with their responsive group and applications.

Polymer	Responsive Group	Applications	Ref.
CS	Amine	Controlled drug release	[145]
P(DEAEMA-co-MMA-co-ABP)	Tertiary amine	Controlled drug release	[31]
PDMAEMA-b-PMMA	Amine	Oil–water separation fabric	[135]
P(NIPAM-co-MAM)	Amine	Smart textile	[47]
PNIPAM-g-CS	Amine	Antibacterial thermoregulating textile	[102]
Polymer containing 2VP	Pyridine	Smart textile, oil–water separation	[134]
UA-co-VTMS	Carboxyl	Oil–water separation fabric	[136]

CS: chitosan; P(DEAEMA-co-MMA-co-ABP): poly(N, N′-diethylamino ethyl methacrylate-co-methyl methacrylate-co-acryloyl benzophenone); PDMAEMA-b-PMMA: poly(2-(dimethylamino)ethyl methacrylate)-b-poly(methyl methacrylate); P(NIPAM–co-MAM): poly(N-isopropylacrylamide-co-methacrylamide); PNIPAM-g-CS: poly(N-isopropyl acrylamide)-g-chitosan; 2VP: 2-vinylpyridine; UA-co-VTMS: (10-undecylenic acid)-co-(vinyltrimethoxysilane).

**Table 4:** Recent examples (2021–2024) from the literature of responsive fibrous structures, emphasizing the material that confers them the ability to respond to a specific stimulus (temperature, light, or pH), along with the methodology of fabrication and potential application.

Stimulus	Material	Structure	Methodology	Application	Ref.
Temperature	N-octadecane	Woven	Microencapsulation of the PCM (N-octadecane) with ODMA-MAA copolymer as shell using suspension-like polymerization; wet spinning of fibers containing PCM, CNTs, graphene and PU; dip-coating of the CNTs-graphene-PCM@PU fibers in a conductive ink of CNTs-graphene/PEDOT:PSS; weaving with the obtained fibers	Smart textiles and wearable electronics	[160]
Temperature	Cholesteryl ester	Nonwoven mat	Formulation of thermochromic LC through the heating at 80–90 °C of ternary mixtures of COC, CB, and CP; electrospinning of blends of LC and polycaprolactone or polystyrene, with chloroform as a solvent	Smart face masks	[38]
Temperature	SFXC and Hallcrest	Knitted and woven fabrics	Coating of yarns (100% PES) with LC inks (SFXC and Hallcrest), by looping the yarns through pulleys and ink barrels, followed by drying and collection; weaving and knitting with the coated yarns	Smart textiles	[22]
Temperature	PNIPAM	Nonwoven	Nonwoven PET scaffold filled with NIPAAm hydrogel; UV irradiation; reactive pore-filling with NIPAAm, MBA as the cross-linker, and APS as the initiator; cross-linking polymerization; PA thin layer obtained by interfacial polymerization; addition of PEG as a pore-forming agent	Thin film composite membranes	[30]
Temperature	Cratin and Hytreal (polymers with different coefficient of thermal expansion)	Nonwoven battings	Side-by-side melt coextrusion of Crastin and Hytrel to form a bicomponent fiber (50–50 composition), where two melt-pump-controlled extruders with pineapple mixers are used for melting the resins, with temperature zones maintained at 230, 240, 250 and 260 °C; fibers transformed into nonwoven battings	Wearable insulation	[162]
Temperature	PEO–PPO–PEO	Nonwoven membrane	Preparation of LLC by mixing and centrifugation of PEO-PPO-PEO, APS, nBA and EGDMA; LLC sandwiched between Mylar films and glass plates; casting on nonwoven polyester sheet under pressure and thermal polymerization at 65 °C	Ultrafiltration membrane	[49]
Temperature	Wool fibers	Knitted fabric	Descaling of raw wool fibers using nanoparticles of calcium carbonate, sodium hypochlorite, and hydrochloric acid; production of double-plied ring-spun dyed woollen yarns using conventional yarn and dyeing technologies; fabrication of single jersey, tuck knit, miss knit, and double-knit structures in a flat-bed knitting machine	Smart textiles	[163]
Temperature	PHA, MDI and BDO	Knitted fabric	Melt spinning of SMP filament using PHA, MDI and BDO; production of knitted fabric using SMP filament and PES yarn on a V-bed knitting machine; shape memory behavior programming by heating up to the transition temperature, deforming up to the required strain, cooling to fix the temporary deformation below 20 °C, releasing the strain, and re-heating to activate the recovery process	Smart textiles	[161]
Temperature	SBMA	Knitted fabric	Spray coating of one side of a knitted cotton fabric with a solution containing a thermo-responsive polymer (SBMA), MBA and TPO-Li; covering of the coated fabric with a tape mask with hole patterns, followed by UV cross-linking	Smart textiles	[105]
Temperature	Bio-based low-melting-point polyamide	Woven 3D fabric	Copolymerization of PA11coPA1218 using the monomers 11-aminoundecanoic acid, 1,12-diaminododecane, and octadecanedioic acid, and sodium hypophosphite monohydrate as a catalyst; surface treatment of starch particles with OSA to make them compatible with the biopolyamide matrix; melt-blending of OSA-treated starch with PA11coPA1218; production of filaments from the copolymer and OSA-treated starch composites; coiling and thermosetting of filaments to form actuators; integration of thermo-responsive coiled actuators into a woven 3D fabric comprised of 100% cotton warp and wefts consisting of linen-tencel, cotton, glow-in-the-dark yarn, and actuators	Smart textiles	[164]
Temperature	PLA and PU	Woven fabric	Wet spinning of PLA/PU/GO fibers using PLA, PU, GO and DMF as solvent; weaving of the fibers using the plain weaving method; spray-coating of one side of the fibers with AgNWs; programming of the SMP textile by stretching deformation at 80 °C, cooling to room temperature and releasing of the stretch force	Smart textiles	[32]
Temperature and light	PNIPAM and GONPs	Nonwoven fiber mat	Synthesis of P(NIPAm-co-HEA-co-AA) microgels by precipitation polymerization of NIPAm, AA, and HEA in water, using SDS as surfactant and BIS as cross-linker; synthesis of P(NIPAm-co-BPAm) via free radical polymerization using NIPAm and BPAm; synthesis of GO sheets and GONPs from graphite; fabrication of GO NWFs via sequential deposition and UV photo-cross-linking of colloidal suspensions of P(NIPAM-co-HEA-co-AA) microgels, P(NIPAM-co-BPAm) and GONPs, onto PP NWFs	Membranes for processing temperature-sensitive bioactive ingredients or remote-controlled bioreactors	[23]
Temperature	PNIPAM and PDMAPS	Woven fabric	Preparation of LCST polymer coating solutions by mixing monomers (NIPAM), EGDMA, and initiator (DEAP) in ethanol, and of UCST polymer coating solutions by mixing monomers (DMAPS), EGDMA and Irgacure D-2959 in DI water/trifluoroethanol; hydrophobic monomer (LMA) or hydrophilic monomer (HEMA) added to the LCST and UCST polymer coating solutions; dipping of cotton yarns in the polymer coating solutions; weaving of textiles with LCST warp yarns, hydrophobic PET yarns, and UCST weft yarns	Smart textiles	[98]
Temperature	LA	Nanofibers	Preparation of ZnO@MWCNT composite materials by dispersion of MWCNTs in deionized water, mixing with Zn(NO_3_)·6H_2_O, and reaction at 140 °C; preparation of phase change nanofibers by electrospinning using LA as a PCM, PVA as a supporting material, SDS as an emulsifier, and ZnO@MWCNT for thermal conductivity; coating of the resultant nanofibers with polydimethylsiloxane and a curing agent, to obtain the resultant hydrophobic, self-cleaning, thermoregulated nanofibers	Wearable systems and protective fabrics	[57]
Temperature and pH	P(NIPAM-co-MAM)	Woven fabric	Synthesis of P(NIPAM-co-MAM) through free radical addition polymerization, using NIPAM and MAM as monomers, at 80 °C; application of the P(NIPAM-co-MAM) copolymer to a 100% cotton plain weaved fabric using a double-bath impregnation method, with the first bath containing the BTCA cross-linker and the SHP catalyst, and the second bath containing an aqueous polymer solution; drying and curing of the fabric	Smart textile	[47]
Temperature	PHEVAH	Woven fabric	VCL monomer was hydrolyzed with EG to produce HEVAH; synthesis of PHEVAH through free radical polymerization, using HEVAH and AIBN in toluene; application of the PHEVAH to a 100% cotton plain weaved fabric using a double bath impregnation, with the first bath containing the BTCA cross-linker and the SHP catalyst, and the second bath containing a polymer solution; drying and curing of the fabric	Smart textile	[43]
Temperature	P(MEO_2_MA-co-OEGMA_500_	Fabric	Graft copolymerization of a cotton fabric with TMSPMA; grafting of P(MEO_2_MA-co-OEGMA_500_) from TMSPMA-cotton using free-radical polymerization, at 37 °C, using HRP, the monomers MEO_2_MA and OEGMA_500_, ACAC, ethanol/phosphate buffer and H_2_O_2_; washing and air-drying of the fabric	Smart textile	[101]
Temperature	PDMAPS	Woven fabric	BN and KH-570 were applied to a twill patterned cotton fabric to improve its hydrophobicity and reactivity; temperature-responsive cotton fabric prepared enzymatically through HRP-catalyzed graft polymerization of PDMAPS, at 37 °C, using HRP, H_2_O_2,_ ACAC, DMAPS, MBA, and phosphate buffer; washing and air-drying of the fabric	Smart oil–water separation fabric	[99]
pH	Chitosan and pectin	Nanofibrous films	Electrospinning of solutions with different combinations of polymers (chitosan and pectin), and HPγCD; preparation of inclusion complexes of curcumin and HPγCD using the freeze-drying method; incorporation of the complexes into chitosan/pectin systems, followed by electrospinning	Biomedical	[159]
pH	P(DEAEMA-co-MMA-co-ABP) (same as PDMA)	Nanofiber mats	Synthesis of PDMA through radical polymerization, at 70 °C, using DEAEMA and MMA as monomers, ABP as cross-linking agents, and AIBN as the thermal initiator, followed by dialysis; electrospinning of solutions containing PDMA, DMF, THF and amoxicillin; UV irradiation and drying of the nanofiber mats	Biomedical	[31]

ABP: 4-acryloyl benzophenone; ACAC: acetylacetone; APS: ammonium persulfate; BDO: 1,4-butanediol; BN: boron nitride; BTCA: 1,2,3,4-butanetetracarboxylic acid; CB: cholesteryl benzoate; COC: cholesteryl oleyl carbonate; CP: cholesteryl pelargonate; DEAP: 2,2-diethoxyacetophenone; DMAPS: N,N-dimethyl (methacryloylethyl) ammonium propane sulfonate; EG: ethylene glycol; EGDMA: ethylene glycol dimethacrylate; HEMA: 2-hydroxyethylmethacrylate; HEVAH: 2-hydroxyethyl-6-(vinyl amino)hexanoate; HPγCD: hydroxypropyl-γ-cyclodextrin; HRP: horseradish peroxidase; Irgacure D-2959: 2-hydroxy-4-(2-hydroxyethoxy)-2-methylpropiophenone; KH-570: 3-(trimethoxysilyl) propyl methacrylate; LA: lauric acid; LC: liquid crystal; LLC: lyotropic liquid crystals; LMA: laurylmethacrylate; MBA: N,N’-methylene bis(acrylamide); MDI: 4,4′-diphenylmethane diisocyanate; nBA: n-butyl acrylate; NWF: nonwoven fiber mat; ODMA-MAA: n-octadecyl methacrylate- methacrylic acid; OSA: octadecenyl succinic anhydride; PHA: poly (1,6-hexanediol adipate); PHEVAH: poly(2-hydroxyethyl-6-(vinyl amino)hexanoate); P(MEO_2_MA-co-OEGMA_500_: poly(diethylene glycol monomethyl ether methacrylate-co- poly(ethylene glycol) methyl ether methacrylate; PVA: poly(vinyl alcohol); SBMA: [2-(methacryloyloxy)ethyl]dimethyl-(3-sulfopropyl); SDS: sodium dodecyl sulphate; SHP: sodium hypophosphite; THF: tetrahydrofuran; TMSPMA: 3-(trimethoxysilyl)propyl methacrylate; TPO-Li: lithium phenyl(2,4,6-trimethylbenzoyl)phosphinate; VCL: N-vinyl caprolactam.
